# Nanoscale patterning of metal nanoparticle distribution in glasses

**DOI:** 10.1186/1556-276X-8-260

**Published:** 2013-06-01

**Authors:** Ivan S Sinev, Mihail I Petrov, Anton K Samusev, Viktoria V Rutckaia, Andrey A Lipovskii

**Affiliations:** 1, National Research University of Information Technologies, Mechanics and Optics, St. Petersburg, 197101, Russia; 2 Department of Physics and Mathematics, University of Eastern Finland, PO Box-111Joensuu, 80101, Finland; 3, St. Petersburg Academic University, St. Petersburg, 194021, Russia; 4, St. Petersburg State Polytechnical University, St. Petersburg, 195251, Russia

**Keywords:** Glass-metal nanocomposites, Near-field scanning optical microscopy, Electric field imprinting, Nanostrips

## Abstract

We show that electric field imprinting technique allows for patterning of metal nanoparticles in the glass matrix at the subwavelength scale. The formation of glass-metal nanocomposite strips with a width down to 150 nm is demonstrated. The results of near-field microscopy of imprinted patterns are in good agreement with the performed numerical modeling. Atomic force microscopy reveals that imprinting also results in the formation of nanoscale surface profile with the height going down with the decrease of the strip width. The experiments prove the applicability of this technique for the fabrication of nanoscale plasmonic components.

## Background

Nowadays, plasmonic materials and structures are the subject of wide-scale studies. In addition to metals, new materials like wide bandgap semiconductors [1,2] and glass-metal nanocomposites (GMN) [3-5], that are glasses embedded with metal nanoparticles, have recently been implemented in plasmonics. Since the dielectric function and, consequently, the propagation of surface plasmon polariton modes in the latter materials can be controlled by varying the volume fraction, size, and type of metal inclusions [5-7], the flexibility of GMN makes them attractive for plasmonics.

The required dimensions of the majority of plasmonic structures [8-10] are in tens of nanometers scale, which compels the use electron beam lithography (EBL) in their fabrication. That is why the search for an alternative cost-effective technique for their manufacturing is of interest.

In [10], nanoimprint lithography was proposed for the fabrication of plasmonic circuits based on metal films, and the usability of electric field imprinting (EFI) process for nanostructuring of GMN has recently been shown [11]. Electric field imprinting of GMN is based on electric-field-assisted dissolution [12-15] (EFAD) of nanoparticles in glass matrix at elevated temperature. This is to control their spatial distribution via application of DC voltage to the GMN using a structured electrode (stamp). The imprinting enables multiple replication of the stamp image to GMN [14,16], that is, mass fabrication of GMN structures. This paper is focused on the characterization of the resolution of GMN EFI using atomic force microscopy (AFM) and scanning near-field optical microscopy (SNOM).

## Methods

Silver-based GMN sample was prepared in a plate of commercial 1-mm thick soda-lime glass using silver-to-sodium ion exchange followed by hydrogen-assisted reduction of silver ions and metal clustering as it was reported elsewhere [17]. According to the results of our previous studies [17], after such processing, the vast majority of the formed silver nanoparticles is located within 200- to 300-nm layer buried under the sample surface at the depth of approximately 100 nm, the diameter of the nanoparticles being around 4 nm. We characterized optical extinction of the sample with optical absorption spectroscopy. The spectra were measured with UV-vis Specord 50 spectrometer (Analytyk Jena, Konrad-Zuse-Strasse, Jena, Germany).

To find the linewidth achievable in the EFI, a profiled glassy carbon [18] stamp with the set of 350-nm deep grooves of 100, 150, 200, 250, 300, 350, 400, 450, 500, and 600 nm in width was fabricated with EBL. The distance between the grooves was equal to 2 *μ*m. The widths and depths of the grooves were checked with scanning electron microscopy (SEM), Zeiss Leo 1550 Field Emission Scanning Electron Microscope (Carl Zeiss Microscopy GmbH, Carl-Zeiss-Strasse, Oberkochen Germany). The stamp was used as the anode in the EFI of both the GMN sample and the plate of virgin glass. The imprinting was carried out at 250°C under 600 V DC.

The imprinted structure was studied using AFM and SNOM techniques using AIST-NT SmartSPM scanning probe microscope and AIST-NT CombiScope Scanning Probe Microscope with optical fiber probe (AIST NT Inc., Novato, CA USA). Numerical modelling was carried out using COMSOL Multiphysics®; package (COMSOL, Inc., Burlington, MA, USA).

## Results and discussion

The measured optical spectrum of the GMN exhibits strong surface plasmon resonance (SPR) absorption centered at 415 nm, and the SPR peak drops after the electric field imprinting (see Figure 1a). The observed blueshift of the SPR peak after the EFI process can be explained by two effects. On one hand, only the most deep-lying nanoparticles can survive after the process of EFI; since they are generally smaller and are surrounded by glass matrix with lower refractive index, the SPR peak exhibits the blueshift. On the other hand, the process of poling of the glass [15] concurrent with EFI decreases the refractive index of the glass matrix due to the evacuation of alkali and silver ions, which also blueshifts the SPR peak.

**Figre 1:**
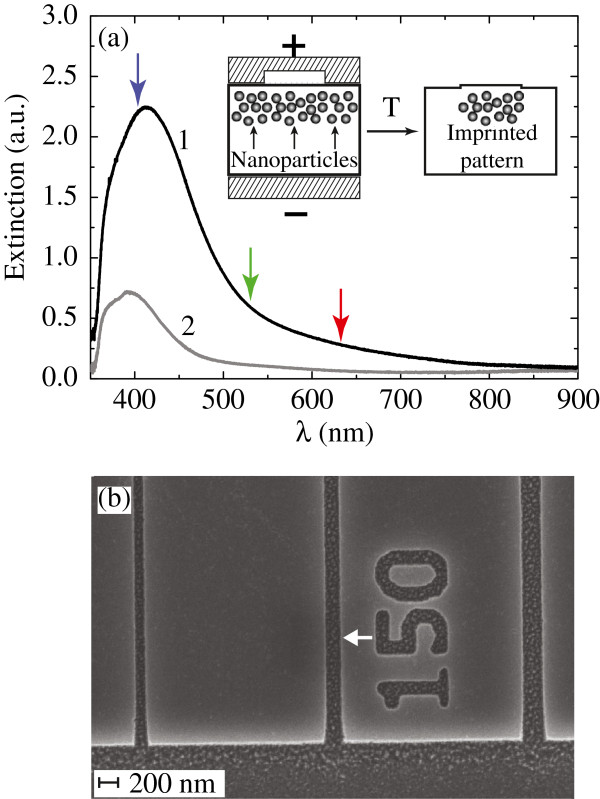
**Extinction spectrum of the GMN and SEM image of the stamp.** (**a**) Extinction spectra of the GMN before (1) and after (2) the imprinting; the wavelengths of lasers used in the near-field experiments are marked with arrows: 633 (red arrow), 532 (green arrow), and 405 nm (violet arrow). The process of imprinting is schematically illustrated in the inset. (**b**) SEM image of the part of glassy carbon stamp used as a positive electrode for imprinting; first three grooves of 100-, 150-, and 200-nm linewidths are shown. The white arrow points to 150 nm groove.

The poling of GMN using the stamp, scanning electron image of a part of which is shown in Figure 1b, has resulted in the dissolution of silver nanoparticles everywhere except the regions beneath the stamp grooves, that is in the formation of GMN strips (see the inset in Figure 1a). In the virgin glass, the imprinting resulted in poling of the glass [15] except the strips beneath the stamp grooves.

The structure imprinted with the stamp is schematically depicted in Figure 2a. The results of the AFM characterization of the imprinted GMN are shown in Figure 2b. Here, one can see that formed surface humps replicate the profile of the used stamp [15,19]. The surface profiling is caused by the relaxation of volume defects generated in the glass matrix after the evacuation of alkali ions from the subanodic region towards the cathode in the course of EFAD [14,15]. The subsidence process is suppressed under the stamp grooves where neither alkali evacuation nor nanoparticle dissolution occurs. It is worth noting that the profile heights measured in the imprinted glass and GMN are of the same order, since the dissolution of the nanoparticles results in the formation of voids coinciding in size with the dissolved particles [20], and the relaxation is related only to the alkali evacuation.

**Figre 2:**
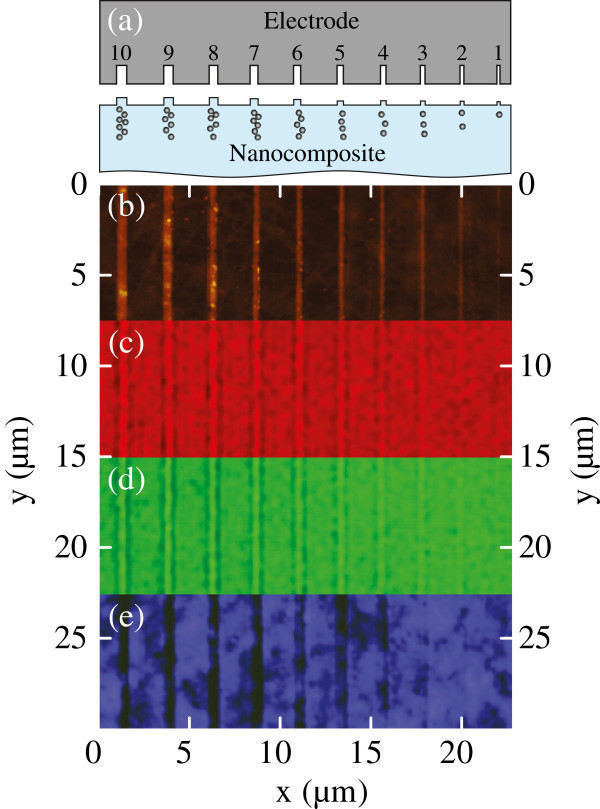
**Imprinted structure and the results of the AFM and SNOM characterization of the imprinted GMN.** (**a**) Scheme of the stamp and the sample surface after the EFI process. The stamp grooves of 100, 150, 200, 250, 300, 350, 400, 450, 500, and 600 nm in width and corresponding imprinted strips are marked with numbers from 1 to 10, respectively. **(b)** AFM of the composite sample surface after the EFI process. Quantitative data is presented in the next figures. Near-field images of the sample at three different excitation wavelengths: (**c**) 633, (**d**) 532, and (**e**) 405 nm.

The results of the AFM measurements averaged along the imprinted strips (see Figure 3, bottom) indicate that the increase in the grooves width up to 500 to 600 nm results in the increase of the hump height up to the value of 45 to 50 nm. For wider strips, the height stays constant. The humps width and height are mainly related through the concurrence of normal and lateral components of ionic fluxes [21] stimulated by local electric field in the vicinity of profiled anode. Additionally, diffusional smearing influences the hump width-to-height relation (stronger for narrow strips).

**Figre 3:**
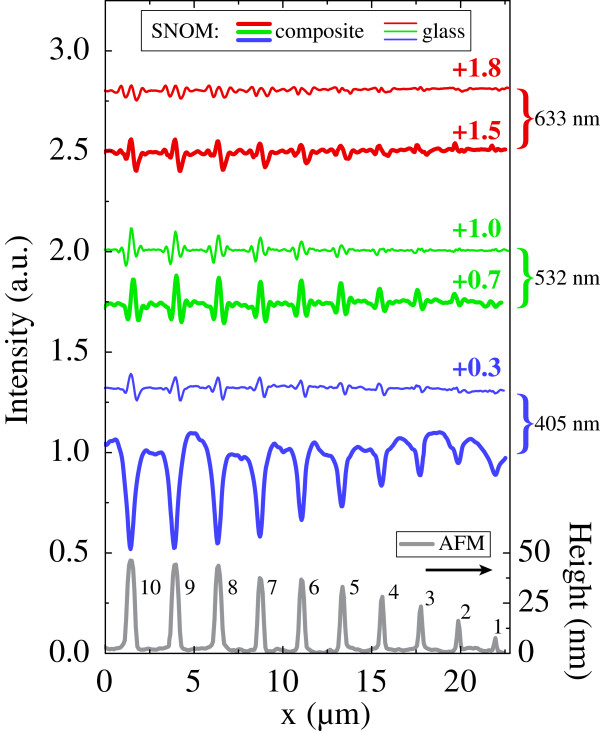
**Near-field optical signal profiles of the composite and virgin glass samples.** Near-field optical signal profiles measured in contact mode for composite sample (thick lines) and virgin glass sample (thin lines) both subjected to the EFI process. The results of three different excitation wavelengths are presented. AFM profile of the composite sample surface is shown at the bottom for convenience; marks 1 to 10 correspond to the stamp groove width from 100 to 600 nm as shown in Figure 2a.

Although the hump formation in the virgin glass and in the GMN, as well as the EFAD of nanoparticles in GMN is due to the ionic redistribution under external voltage [22], there is no evidence of their exact correspondence. To characterize the nanoparticle distribution, we resorted to near-field optical microscopy operating in transmission mode (the sample was excited through the objective, and scattered light was collected with fiber probe). The setup allowed us to scan samples both in contact with the surface and in plane scan mode. The latter regime allows scanning within a plane calculated relying on the sample surface with the preselected lift value. In the experiments, the electric field vector of the incident light wave was directed perpendicularly to the imprinted strips. The SNOM measurements of the patterned glass and the GMN sample were carried out at three laser wavelengths: 633 (red), 532 (green), and 405 nm (violet). The optical absorption of GMN for these wavelengths respectively increased, having the resonance at 415 nm (see Figure 1a, the used wavelengths are marked with arrows), while the virgin glass sample absorption varied with probing wavelength very slightly.

The results of 2D scanning of imprinted GMN sample in plane scan mode with 100-nm lift are shown in Figure 2c,d,e. One can see the imprinted structures easily, the optical contrast at the violet wavelength corresponding to the SPR absorption being much stronger than one at green and red wavelengths. The difference in the intensities measured in contact and in plane scan modes was not significant; this could be due to the fact that the layer of nanoparticles in GMN can be buried about 100 nm below the surface [17]. The intensity profiles obtained after averaging of 2D contact mode scans of the imprinted virgin glass and GMN sample along the strips are shown in Figure 3. The measurements of the glass sample at all three wavelengths and the measurements of the GMN sample at red and green wavelengths showed optical signal intensity modulation with maximum amplitude of about 10%. At the same time, the intensity dips with the amplitude up to 50% were observed at the violet laser wavelength which corresponds to the SPR of silver nanoparticles in GMN strips that survived under the stamp grooves. The amplitude of the intensity modulation is constant when the GMN strip width exceeds 500 to 600 nm and decreases with the strip width at all probing wavelengths used.

Generally, the observed modulation could be due to local light absorption in the strips, to the interference of incident light wave with the wave scattered by the surface humps, and to the light wave phase shift difference in poled (out of strips) and unpoled regions of the glass sample. The latter effect may come from the refractive index change in poled glass, which amounts to *Δn*∼−(0.03−0.09) [23]. Basing on close magnitudes of the modulation as well as the shape of the SNOM signal measured on the glass and on the GMN at red (633 nm) and green (532 nm) wavelengths, we can conclude that far from the SPR, where GMN absorption is low and the refractive index of GMN is close to the one of the glass, the registered near-field intensity modulation in GMN and in the glass has the same nature. On the contrary, much stronger intensity modulation is observed at 405 nm (see Figure 3), corresponding to the SPR light absorption, which proves the presence of silver nanoparticles in the strips beneath the stamp grooves. One can see in Figure 3 that relevant signal drop for 150 nm GMN strip is observed; however, we cannot claim imprinting of 100 nm strip as the signal was smeared after the averaging of 2D data. Thus, the formation of surface profile of 100 nm linewidth element was not followed by the modulation of nanoparticle concentration at the same scale.

To interpret the obtained experimental results numerical modelling has been used. The results of near-field intensity calculations at 100-nm distance above the glass plate with GMN strips corresponding to the stamp used in EFI are shown in Figure 4 jointly with the experimental data measured in plane scan mode at the same distance from the surface. The Maxwell-Garnett effective medium approach with filling factor *f*=0.01 was used for the modeling of GMN optical parameters. In the calculations, we used a 300-nm GMN layer buried at 150-nm depth. One can see good correspondence of the experimental data and our modeling. It is worth to highlight that the nanocomposite fill factor was assumed to be the same for all imprinted strips. Thus, the comparison of the model and the experiment bear evidence that even in the 150 nm imprinted strip, the concentration of the nanoparticles is roughly the same as in the initial GMN sample; the lower magnitude of the light modulation as compared to the thicker strips is due to geometrical factor only.

**Figre 4:**
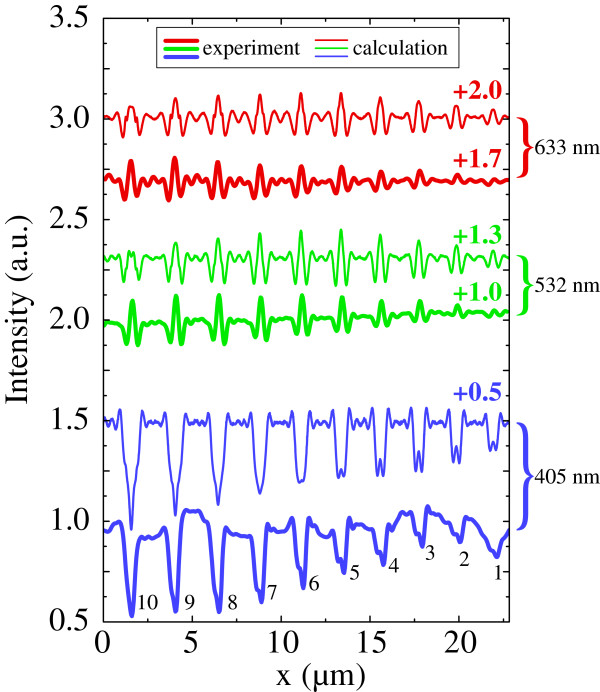
**Results of the experiments and near-field intensity calculations at 100-nm distance above the glass plate.** Optical signal profile measured at the distance of 100 nm above the sample surface (thick lines) and the the square of electric field modulus at the same distance from the sample surface calculated using COMSOL Multiphysics®; (thin lines). The results of the three different wavelengths are presented; marks 1 to 10 correspond to the stamp groove width from 100 to 600 nm as shown in Figure 2a.

## Conclusions

Finally, in this study, we used a scanning near-field optical microscopy to characterize the spatial resolution of the EFI technique applied to the glass-metal nanocomposites. For this purpose, we replicated a set of nanostrips differing in width to the silver-based glass-metal nanocomposite sample using a profiled glassy carbon stamp as the anodic electrode. Our near-field measurements showed significant dependence of optical transmission of the imprinted strips on the excitation wavelength. In contrast to relatively low modulation of optical signal at 633- and 532-nm wavelengths, the transverse scan of the intensity profile at 405 nm contained sharp dips corresponding to the silver nanoparticle surface plasmon resonance absorption in the imprinted strips. Numerical simulations of near-field signal under the assumption that the nanoparticle concentration is equal in all of the strips showed good agreement with our experiment. Finally, this study proved that glass-metal nanocomposite elements with linewidth down to at least 150 nm can be fabricated with electric field imprinting technique.

## Competing interests

The authors declare that they have no competing interest.

## Authors’ contributions

ISS conducted SNOM, AFM, and spectroscopy measurements. AKS supervised the experiments and participated in data processing. MIP developed the models used. VVR prepared the samples from ion exchange until their annealing in hydrogen and performed the numerical calculations. AAL supervised the whole work starting from sample preparation to analysis of data. All authors read and approved the final manuscript.

## Author’s Information

ISS is a Masters degree student of St. Petersburg Academic University and an assistant at the National Research University of Information Technologies, Mechanics and Optics. MIP is a former PhD student of the University of Eastern Finland; he defended the thesis in April 2013. AKS is a PhD degree holder and is a junior research fellow at the National Research University of Information Technologies, Mechanics and Optics; he defended his thesis at Ioffe Institute in December 2011. VVR has graduated from St. Petersburg Academic University in 2012. AAL holds a DrSci degree and Professor positions in St. Petersburg Academic University and St. Petersburg State Polytechnical University.
